# Primary percutaneous coronary intervention with diagnostic catheter in an anomalous origin right coronary artery—a case report

**DOI:** 10.1186/s43044-020-00083-z

**Published:** 2020-08-05

**Authors:** Mohd Iqbal Dar, Aamir Rashid, Mohd Iqbal Wani, Hilal A. Rather, Khursheed A. Khan

**Affiliations:** Department of Cardiology, SKIMS, Soura, Jammu and Kashmir 190011 India

**Keywords:** Anomalous coronary artery, Sinotubular junction, Right coronary artery, Primary percutaneous coronary intervention, Case report

## Abstract

**Background:**

Although rare, the possibility of encountering an anomalous coronary artery is a reality. The outcome of such a procedure is greatly influenced by the awareness of the operator about the anatomical variations and the technique required.

**Case presentation:**

A 50-year-old female patient presented with chest pain. On evaluation, she was found to have an inferior wall myocardial infarction. Left coronary angiography showed non-obstructive disease in the left anterior descending (LAD) and left circumflex artery (LCX). The right coronary artery could not be hooked despite multiple attempts and catheter changes. A non-specific aortic angiogram revealed anomalous origin of the right coronary artery (RCA) above the sinotubular junction on the left side. RCA was hooked with the AL-2 diagnostic catheter, and the percutaneous coronary intervention (PCI) procedure was completed via the same diagnostic catheter.

**Conclusion:**

In a life-threatening difficult situation like acute coronary syndrome with anomalous origin of coronary arteries, PCI can be done using a diagnostic catheter.

## Background

Primary percutaneous coronary intervention continues to be the preferred treatment of acute coronary syndrome worldwide. With the increased penetrance and availability of this treatment, there has been a steady increase in the detection of anatomical variation in the coronary circulation. These anatomical variations especially during an emergency procedure can lead to increase procedure time, radiation exposure, dye use, complications, and overall adverse outcomes. Additionally, these variations can cause tremendous stress to the operators. Here we describe a unique case with such difficulties.

## Case presentation

**Fig. 1:**
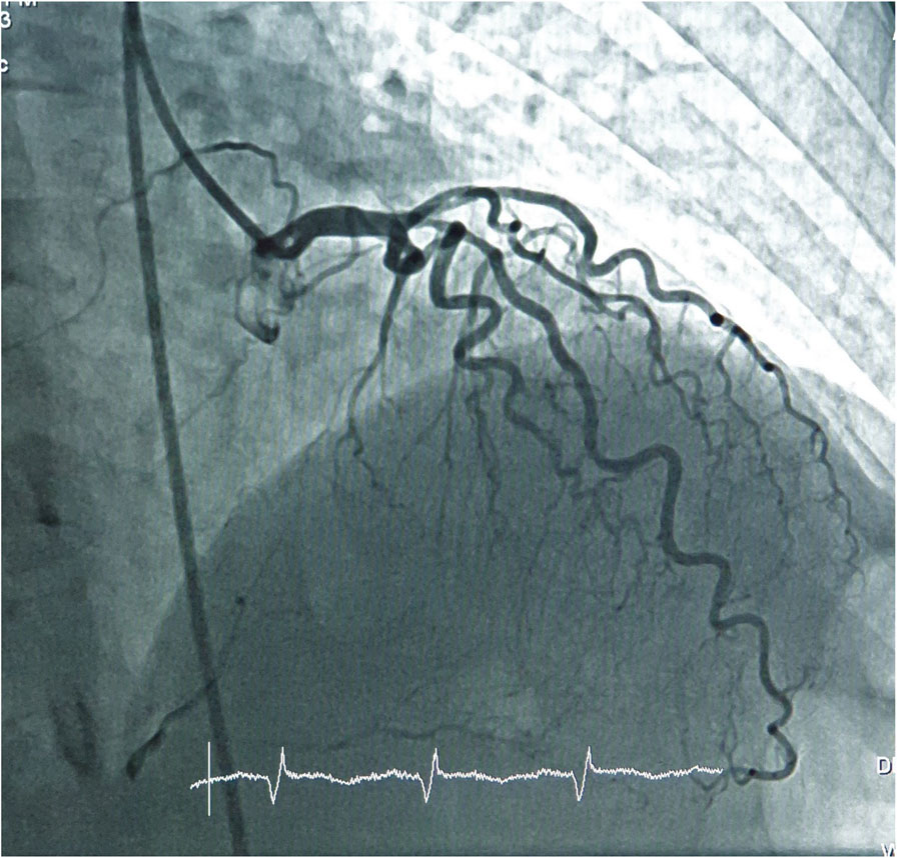
Left side coronary angiogram

A 50-year-old female presented with a 4-h history of acute retrosternal chest pain radiating to the left arm associated with diaphoresis. The patient had a history of hypertension for 10 years and a smoking history of 15 years. There was no any other significant past history. On examination, patient had a pulse rate of 60 beats/min and a blood pressure of 90/60 mmHg. ECG showed an inferior wall myocardial infarction with ST elevation in inferior leads. Echocardiography showed regional wall motion abnormality in the inferior wall with an ejection fraction of 52%. The patient was loaded with aspirin and clopidogrel (600 mg), and the patient was quickly taken for primary PCI via a femoral route with intravenous unfractionated heparin (@100 U/kg followed by additional doses according to activated clotting time) used as an anticoagulant for the procedure. Left-sided angiography taken using a JL 3.5 diagnostic catheter revealed only non-obstructive disease in the proximal left anterior descending artery (LAD) and distal left circumflex coronary artery (LCX). Distal RCA was filling retrogradely from contra-lateral collaterals as shown in Fig. 1. Multiple attempts with manipulation were made with the JR 3.5 diagnostic catheter to hook RCA; however, it was unsuccessful. The catheter was changed to the tiger catheter, Amplatz Right 1, Amplatz left 1 diagnostic catheter and EBU, but these could not identify the ostium of RCA. A non-specific angiogram taken in the ascending aorta revealed RCA ostium to be present anteriorly and high above the sinotubular junction on the left side of the ascending aorta.

**Fig. 2:**
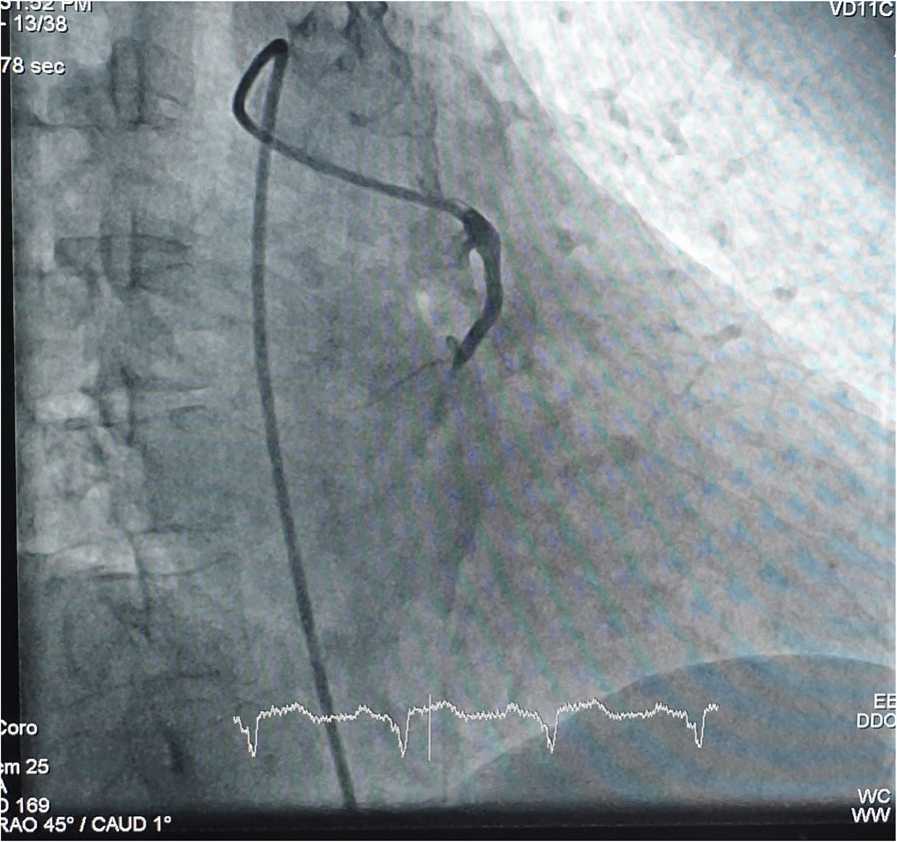
RAO showing anomalous origin and proximally cutoff RCA

**Fig. 3:**
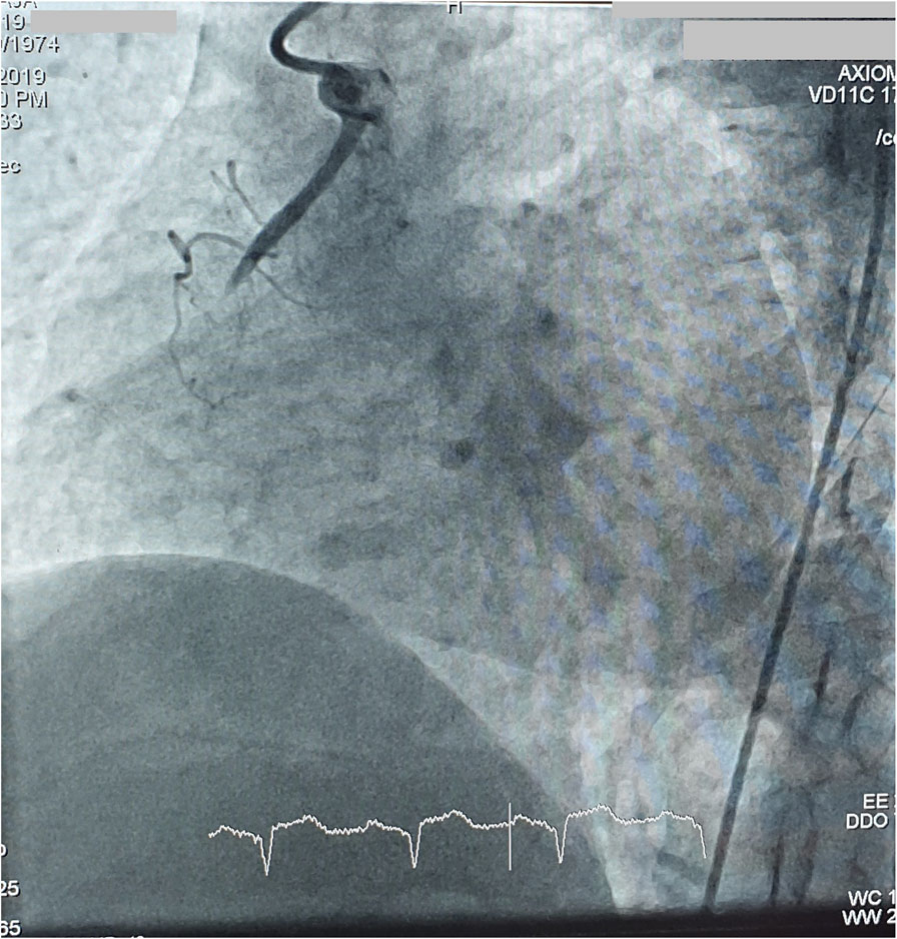
LAO view showing anomalous origin and proximally cutoff RCA

Judkins left diagnostic and guiding, EBU guiding catheter, and AL-1 could not hook the RCA. Finally, RCA was hooked with difficulty with the AL-2 (Medtronic® USA) diagnostic catheter after careful manipulation. A right-sided angiogram revealed RCA cutoff proximally as shown in Figs. 2 and 3.

**Fig. 4:**
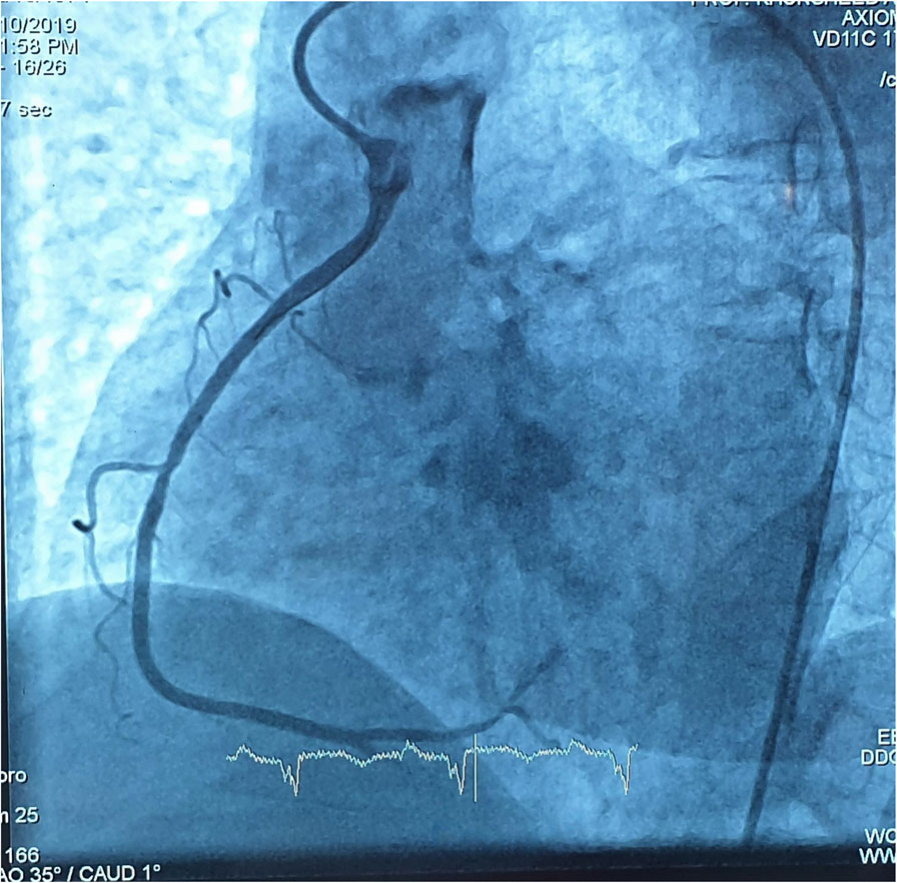
Coronary angiogram showing TIMI 3 flow in RCA after the procedure

In view of the difficult hooking and considering the patient’s condition, the intervention was continued with this diagnostic catheter. The lesion was crossed with a BMW wire, and the lesion dilated with 2 × 12 mm SC balloon at 16 atmospheres which restored the blood flow. A 3 × 20 mm endeavor resolute was deployed at the lesion site at 14 atmospheres and post dilated with 3 × 8 mm NC balloon at 20 atmospheres with TIMI 3 flow in the vessel post-procedure as shown in Fig. 4. The procedure was completed in 1 h and 5 min, fluoroscopy time of 20 min, and the total amount of radiation exposure during the procedure was 816 mGy. The amount of dye used in this procedure was 220 ml. The procedure time, fluoroscopic time, radiation exposure, and dye used were higher than those of a routine RCA PCI procedure. This was significantly due to the use of multiple catheters in an attempt to hook the RCA. The patient was discharged on day 3 from the hospital in a stable condition.

## Discussion

Coronary artery anomalies (CAA) generally affect nearly 1% of the population; however, the estimates vary from 0.3 to 5.6% in different angiographic studies to nearly 1% in routine autopsy. These anomalies vary in the clinical spectrum from potentially fatal to a largely benign condition [1]. Anomalies in the origin of the coronary artery also known as anomalous origin of the coronary artery (AOCA) constitute a small subset of coronary artery anomalies and are seen in about 0.2–1.2% of patients undergoing coronary angiography. The risk associated with AOCA depends upon the anatomical path taken by the artery to its perfusion territory [2]. The association of coronary artery disease (CAD) in CAA is not clear; AOCA has been shown to be associated with an increase incidence of atherosclerotic CAD [3], while no such association could be established in other abnormalities like coronary artery ectasia [4] although most of the patients presented with symptoms of typical angina.

Anomalous origin of the right coronary artery from the left coronary sinus, first described by Yans et al. [5], has a prevalence of 0.026 to 0.255% in different autopsy and coronary angiographic studies [6]. The origin of anomalous right coronary artery in relation to left coronary artery ostium and sinotubular junction is divided into 4 types: type A, origin from the aorta above the sinotubular plane; type B, origin just below the ostium of the left coronary artery (LCA); type C, origin below the sinotubular plane between the midline and the origin of left coronary artery; and type D, origin along the midline of the ascending aorta. These different origins demand a different type of catheter for easy cannulation [2]. Anomalous RCA from left coronary sinus has been shown by multiple studies to be associated with angina, syncope, ventricular tachycardia, and sudden death even in the absence of atherosclerotic lesions [7–9].

Anomalous origin of coronary artery causes significant difficulty in patients requiring PCI. The procedure is usually associated with increased radiation exposure to both the patient and operator, an increase in the amount of dye used, and an increase in the procedure time. There is also an associated increase in the psychological stress to the operator. There have been few reports of primary PCI done in patients with anomalous RCA published so far using guiding catheters. Another study has evaluated the use of diagnostic catheters for PCI in a difficult situation and found it helpful in certain cases [10].

The current case with RCA arising from above the sinotubular junction (type A) has an estimated prevalence of 0.006% [2]. Just more than a dozen cases have been defined in literature so far, and only a few among them with acute coronary syndrome required primary intervention [11–13]. The current case in our view is probably the first case of anomalous origin RCA type A in which primary PCI was done using a diagnostic catheter successfully. Pre- and post-dilatations, balloons could be easily passed without much difficulty through the catheter without dampening the pressure tracing. There was also adequate visualization of the coronary artery on dye injection, although in some studies there has been inadequate opacification of the distal vessel in procedure with diagnostic catheters. There were no other difficulties encountered during the procedure. The use of diagnostic catheters for simple percutaneous coronary intervention has been found to be feasible; however, the use of diagnostic catheters for PCI for complex coronary intervention requiring thrombosuction or bifurcation stenting is not possible due to smaller lumen of the diagnostic catheters [10]. This case is aiming to generate awareness about the different anatomical variations of the coronary artery and its management during an emergency procedure.

## Conclusion

Primary percutaneous coronary intervention with a diagnostic catheter is a viable option in patients with anomalous coronary artery origin in which other catheters fail.

## Data Availability

All the data used in this study is available with the corresponding author.

## Abbreviations

BMW: Balanced middle weight
TIMI: Thrombolysis in myocardial infarction
SC balloon: Semi-compliant balloon
NC: Non-compliant balloon
